# Supplementary material for: Preoperative Home-Based Multimodal Physiotherapy in Patients Scheduled for a Knee Arthroplasty Who Catastrophize About Their Pain: A Randomized Controlled Trial
Source: J Clin Med. 2025 Jan 5;14(1):268. doi: 10.3390/jcm14010268 (PMC11720860; doi:10.3390/jcm14010268)

Supplementary Materials

Figure S1. Evolution of Pain Catastrophizing Scale ratings over time.

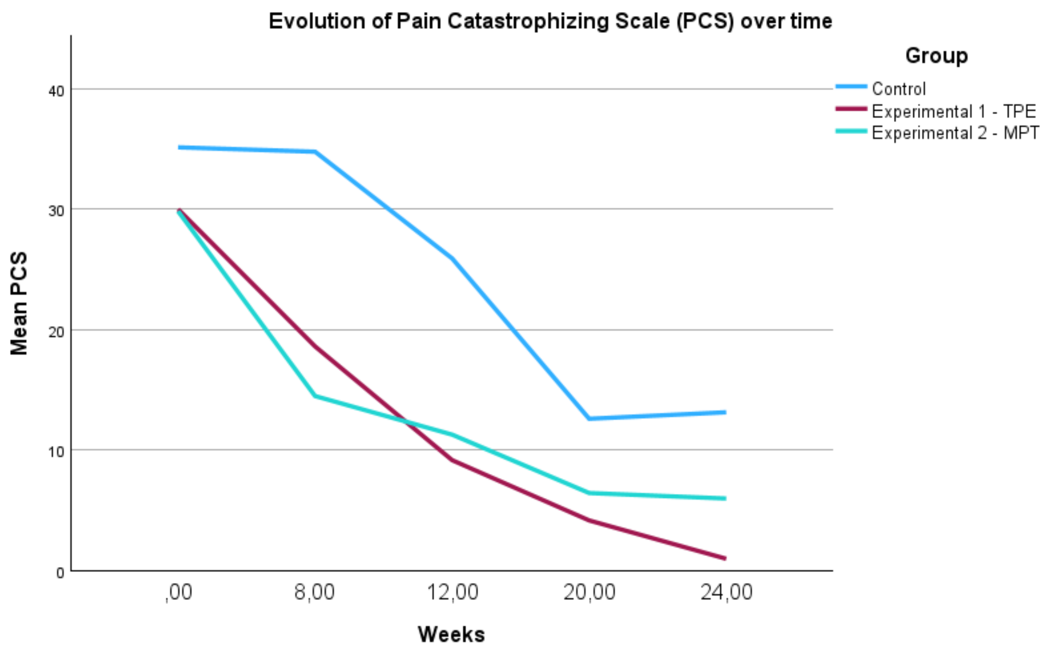

Figure S2. Evolution of Pain Intensity Ratings at Rest Over Time.

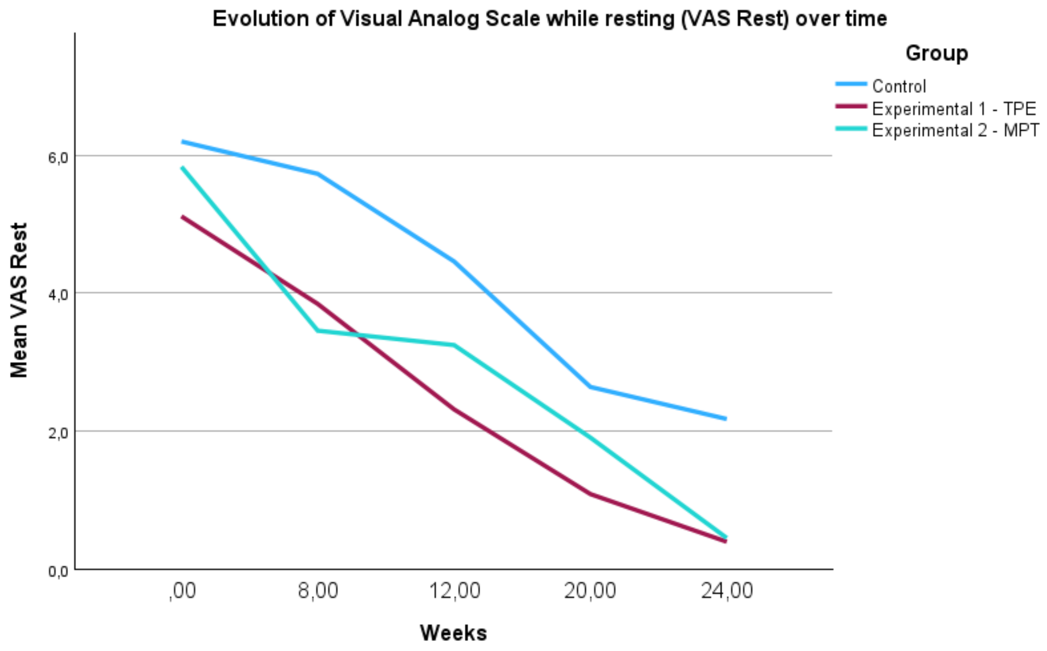

Figure S3. Evolution of Pain Intensity Ratings while Walking Over Time.

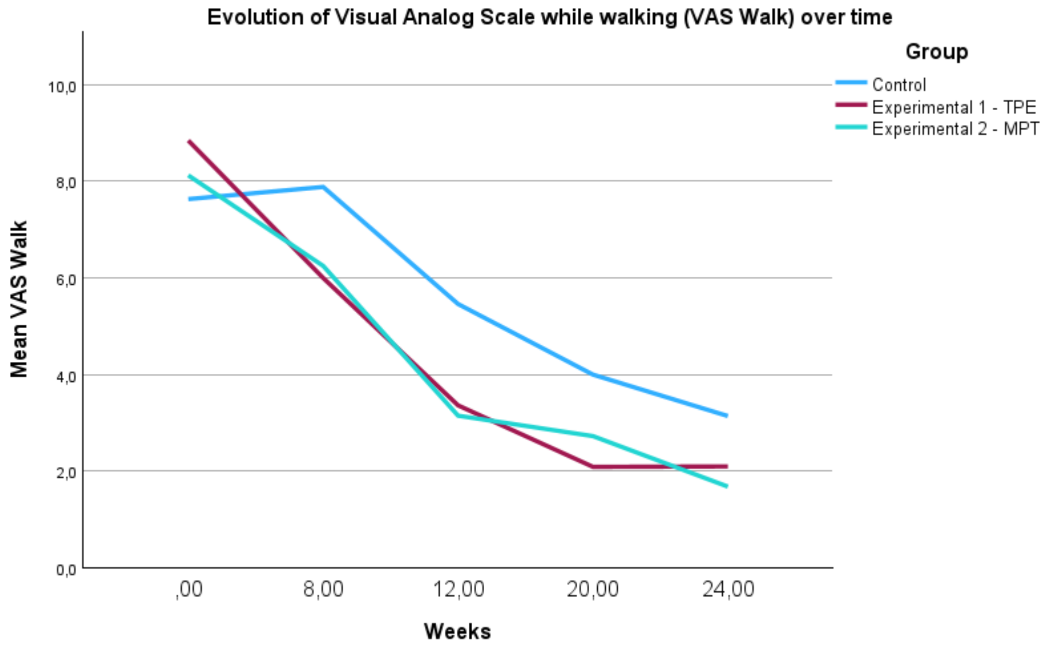

Figure S4. Evolution of Western Ontario and McMaster University Index Pain Subscale Over Time.

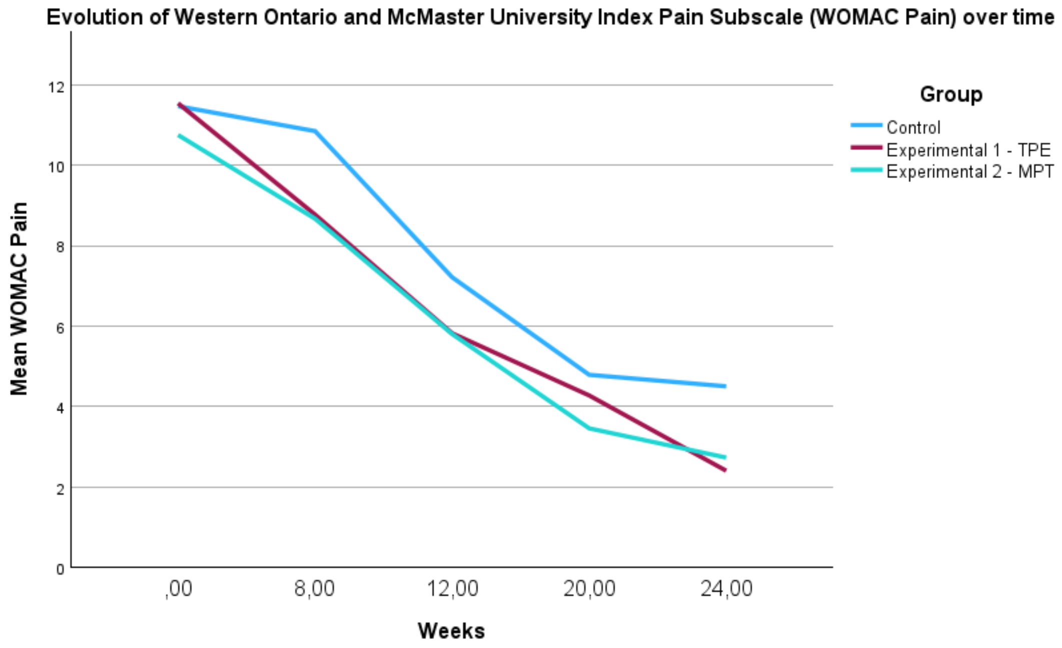

Figure S5. Evolution of Western Ontario and McMaster University Index Total Score Over Time.

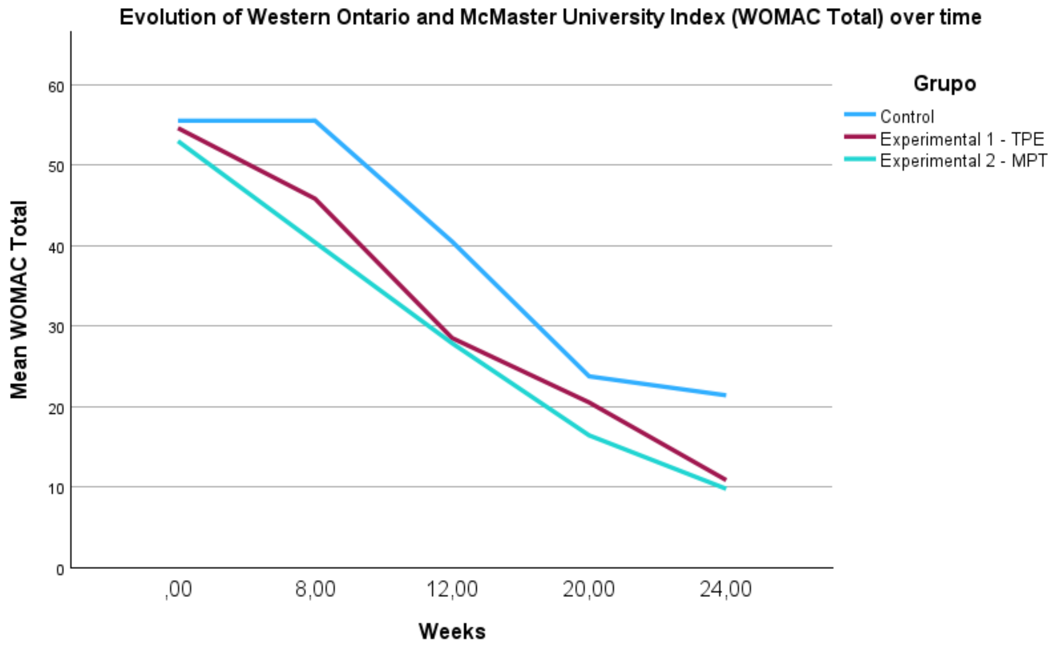

Figure S6. Evolution of Pain-Related Fear of Movement Ratings Over Time, using The Tampa Scale of Kinesiophobia.

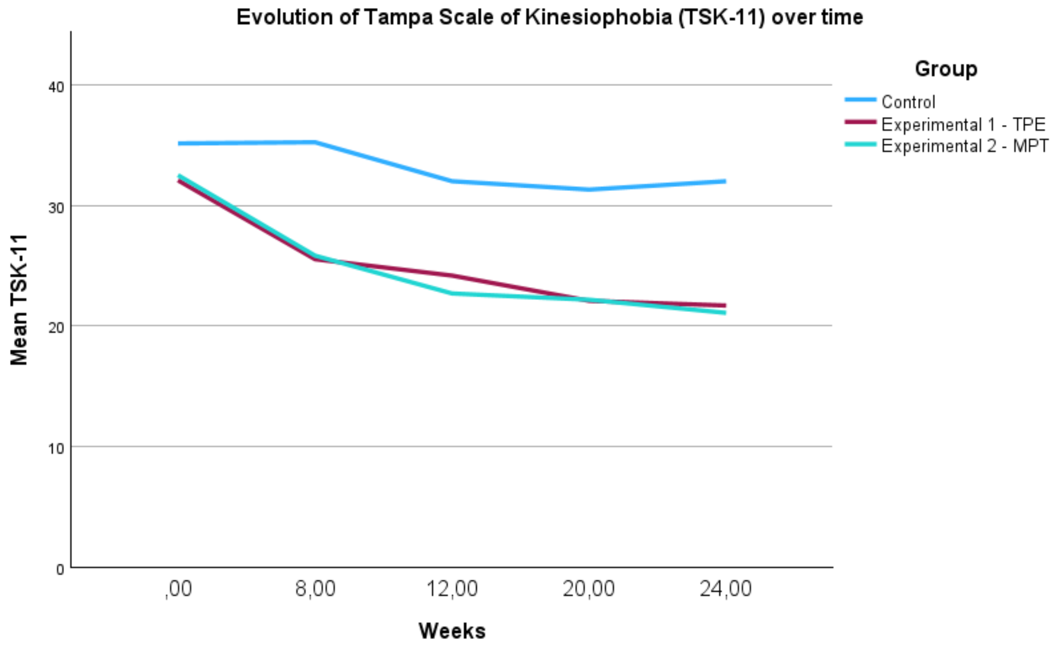

Figure S7. Evolution of Self-Efficacy Ratings Over Time, using Chronic Pain Self-Efficacy Scale.

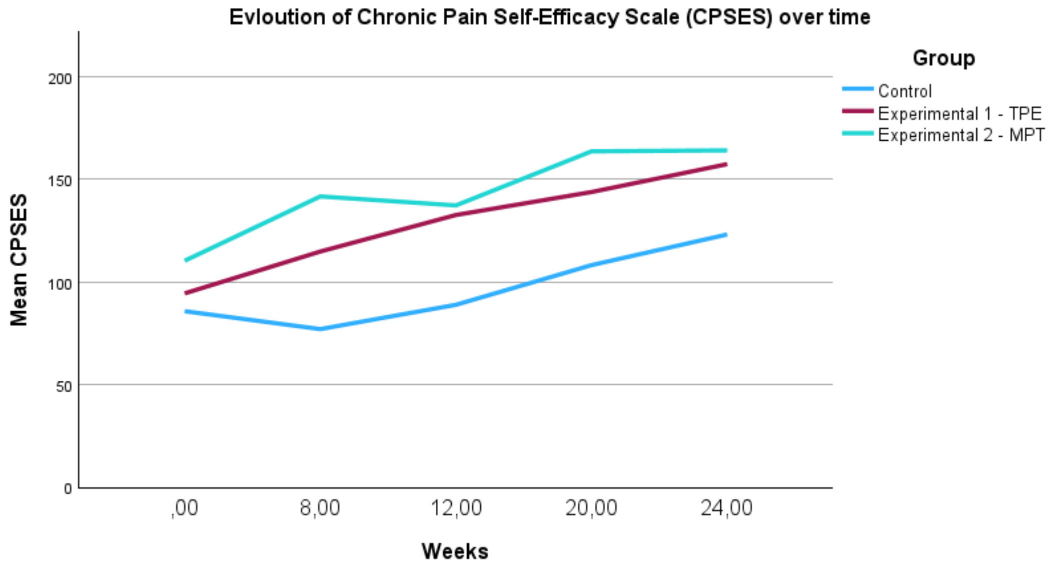

Figure S8. Evolution of Walking Speed Over Time, using The 4-Meters Walking Test.

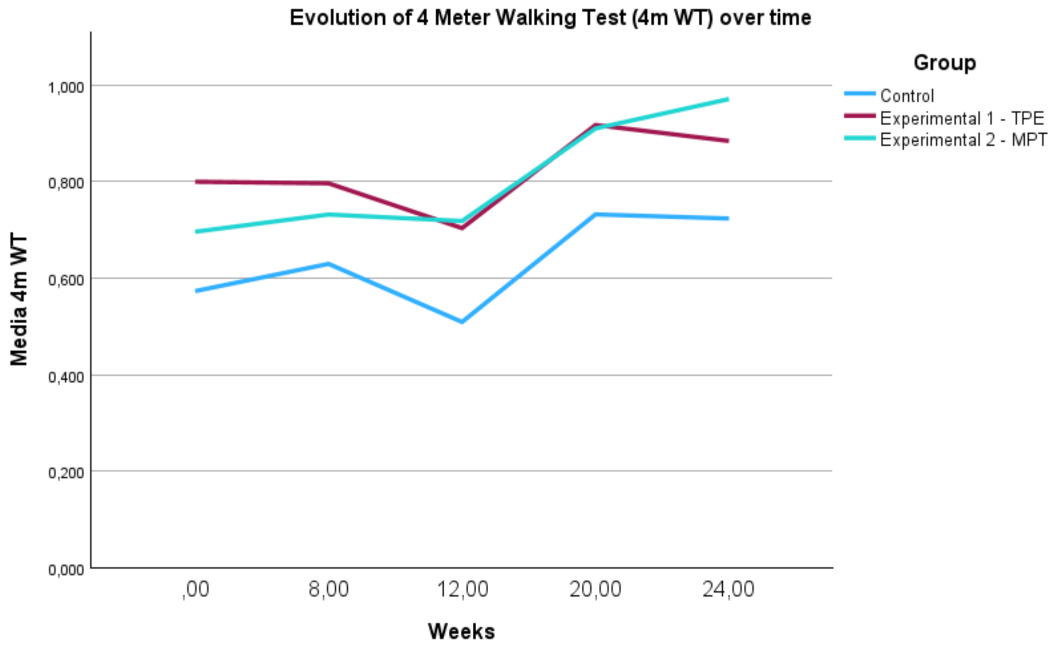

Supplement: Supplementary file 1 [file jcm-14-00268-s001.zip › jcm-3389643-supplementary.pdf]
